# Supplementation of oligosaccharide-based polymer enhanced growth and disease resistance of weaned pigs by modulating intestinal integrity and systemic immunity

**DOI:** 10.1186/s40104-021-00655-2

**Published:** 2022-01-12

**Authors:** Kwangwook Kim, Yijie He, Cynthia Jinno, Lauren Kovanda, Xunde Li, David Bravo, Eric Cox, Yanhong Liu

**Affiliations:** 1 Department of Animal Science, University of California, Davis, CA 95616 USA; 2 School of Veterinary Medicine, University of California, Davis, CA 95616 USA; 3 Pancosma|ADM, 1180 Rolle, Switzerland; 4 Department of Virology, Parasitology and Immunology, Ghent University, 9000 Ghent, Belgium

**Keywords:** Enterotoxigenic *E. coli*, Growth rate, Intestinal barrier function, Oligosaccharide-based polymer, Systemic immunity, Weaned pigs

## Abstract

**Background:**

There is a great demand for antibiotic alternatives to maintain animal health and productivity. The objective of this experiment was to determine the efficacy of dietary supplementation of a blood group A6 type 1 antigen oligosaccharides-based polymer (Coligo) on growth performance, diarrhea severity, intestinal health, and systemic immunity of weaned pigs experimentally infected with an enterotoxigenic *Escherichia coli* (ETEC), when compared with antibiotics.

**Results:**

Pigs in antibiotic carbadox or Coligo treatment groups had greater (*P* < 0.05) body weight on d 5 or d 11 post-inoculation (PI) than pigs in the control group, respectively. Supplementation of antibiotics or Coligo enhanced (*P* < 0.05) feed efficiency from d 0 to 5 PI and reduced (*P* < 0.05) frequency of diarrhea throughout the experiment, compared with pigs in the control group. Supplementation of antibiotics reduced (*P* < 0.05) fecal β-hemolytic coliforms on d 2, 5, and 8 PI. Pigs in antibiotics or Coligo groups had reduced (*P* < 0.05) neutrophil counts and serum haptoglobin concentration compared to pigs in the control group on d 2 and 5 PI. Pigs in Coligo had reduced (*P* < 0.05) total coliforms in mesenteric lymph nodes on d 5 and 11 PI, whereas pigs in antibiotics or Coligo groups had reduced (*P* < 0.05) total coliforms in spleen on d 11 PI compared with pigs in the control group. On d 5 PI, pigs in the Coligo group had greater (*P* < 0.05) gene expression of *ZO1* in jejunal mucosa, but less (*P* < 0.05) mRNA expression of *IL1B*, *IL6*, and *TNF* in ileal mucosa, in comparison with pigs in the control group. Supplementation of antibiotics enhanced (*P* < 0.05) the gene expression of *OCLN* in jejunal mucosa but decreased (*P* < 0.05) *IL1B* and *IL6* gene expression in ileal mucosa, compared with the control. On d 11 PI, supplementation of antibiotics or Coligo up-regulated (*P* < 0.05) gene expression of *CLDN1* in jejunal mucosa, but Coligo reduced (*P* < 0.05) *IL6* gene expression in ileal mucosa compared to pigs in the control group.

**Conclusions:**

Supplementation of Coligo improved growth performance, alleviated diarrhea severity, and enhanced gut health in weaned pigs infected with ETEC F18 in a manner similar to in-feed antibiotics.

**Supplementary Information:**

The online version contains supplementary material available at 10.1186/s40104-021-00655-2.

## Background

Enterotoxigenic *E. coli* (ETEC) strains expressing F4 or F18 fimbriae are major causes of post-weaning diarrhea in nursery pigs [1]. Attachment of ETEC to the specific receptors on intestinal epithelium leads to colonization and secretion of enterotoxins, resulting in secretory diarrhea in weanling pigs [2]. To prevent post-weaning diarrhea and improve production of pigs, antibiotics were commonly added to the diet over the past decades. However, frequent use of in-feed antibiotics in livestock production has been shown to contribute to the increased prevalence of antibiotic-resistant bacteria and raised public health concerns [3]. With these issues, the Food and Drug Administration (FDA) banned the use of in-feed antibiotics for growth promoting purposes in livestock production in the U.S. [4], thus alternative nutritional strategies are highly demanded to enhance disease resistance and production of weanling pigs.

Many nutritional approaches have been applied to prevent post-weaning diarrhea associated with ETEC and enhance the production of pigs. Among these, the prevention of bacterial attachment to the small intestine is one of the most effective defense strategies against ETEC infection [5]. Oligosaccharides have been reported to possess ETEC receptor activity for bacterial adhesions [6, 7]. Especially, N-acetylgalactosamine (GalNAc) containing glycans that could enhance the binding affinity of various ETEC strains, including K99 [8], F4 [9], and F18 [10–12]. Coddens et al. [11] identified the blood group H type 1 determinant (Fuca2Galß3GlcNAc) as the minimal binding epitope of F18 fimbriae. Based on that, an optimal binding epitope was created by adding the terminal 3-linked galactose or *N*-acetylgalactosamine of the blood group B type 1 determinant (Galα3(Fucα2)Galß3GlcNAc) and the blood group A type 1 determinant (GalNAcα3(Fucα2)- Galß3GlcNAc). The purified soluble blood group oligosaccharides were able to greatly reduce binding of F18 positive *E. coli* to intestinal villi of F18 receptor-positive pigs in concentrations of 1 to 10 mg/mL [11]. Moreover, multimerizing blood group A on human serum albumin reduced the amount of blood group oligosaccharides needed 1000 times [13]. Therefore, multimerizing the blood group A oligosaccharides may efficiently prevent enterotoxin-induced secretory diarrhea. Recently, grafted polymers that combine multiple substances have been proposed for their potential synergistic effects on preventing human and animal diseases [14, 15]. Epsilon-poly-lysin (ε-PL) has been reported to use as a carrier in the membrane transport of proteins and drugs [16]. Due to its excellent heat stability, biodegradability, and lack of toxicity, ε-PL has generally been regarded as safe status (GRAS) and been interested in the food and medicine industries as a delivery vehicle targeting the desired location [17]. Thus, the combination of blood group oligosaccharides and ε-PL may enhance the resistance of pigs against F18 ETEC infection by inhibiting bacterial attachment and/or directly killing bacteria. The overall objective of this study was to investigate the efficacy of blood group A6 type 1-based polymer on gut integrity and disease resistance of weanling pigs challenged with F18 ETEC.

## Materials and methods

### Animals, housing, experimental design, and diet

A total of 48 weanling pigs (crossbred; initial body weight (BW): 7.23 ± 1.14 kg; 21 days old) with an equal number of gilts and barrows were used in this study. They were selected from the Swine Teaching and Research Center at the University of California, Davis. The sows and piglets used in this experiment did not receive *E. coli* vaccines, antibiotic injections, or antibiotics in creep feed. Before weaning, feces were collected from sows and all their piglets destined for this study to verify the absence of β-hemolytic *E. coli*. The F18 ETEC receptor status was also tested based on the methods of Kreuzer et al. [18], and all piglets used in this study were susceptible to F18 ETEC.

After weaning, all pigs were randomly assigned to one of the four dietary treatments in a randomized complete block design with body weight within sex and litter as the blocks and pig as the experimental unit. There were 12 replicate pigs per treatment. Pigs were individually housed (pen size: 0.61m × 1.22 m) in environmental control rooms at the Cole Facility at the University of California, Davis for 18 d, including 7 d before and 11 d after the first F18 ETEC challenge (d 0). The piglets had ad libitum access to feed and water. Environmental enrichment was provided for each pig. The light was on at 07:00 h and off at 19:00 h daily in the environmental control rooms.

The 4 dietary treatments included: 1) Positive control: control diet; 2) Low dose oligosaccharide-based polymer (LOW): control diet supplemented with 10 mg/kg oligosaccharide-based polymer active substance (Coligo); 3) High dose oligosaccharide-based polymer (HIGH): control diet supplemented with 20 mg/kg oligosaccharide-based polymer active substance (Coligo); and 4) CAR: control diet supplemented with 50 mg/kg carbadox. Spray-dried plasma and high levels of zinc oxide exceeding recommendation and normal practice were not included in the diets. The experimental diets were fed to pigs throughout the experiment. Oligosaccharide-based polymer active substance was a glycoconjugate composed of blood group A6 type 1 antigen oligosaccharides grafted on a single peptide of epsilon-poly-lysine. Coligo was designed and synthesized by Elicityl (France) in cooperation with Ghent University (Dr. Eric Cox’s laboratory) and was provided by Pancosma (Geneva, Switzerland). The mean rate of conjugation is 15 mol of oligosaccharide for 1 mol of ε-PL. The oligosaccharide part represents 25% of the molecular weight of the oligosaccharide-based polymer. All diets were formulated to meet pig nutritional requirements (Table 1) [19] and provided as mash form throughout the experiment.

**Table 1:** Ingredient compositions of experimental diets^1^

Ingredient, %	Control diet
Corn	44.51
Dried whey	15.00
Soybean meal	14.00
Fish meal	10.00
Soy protein concentrate	7.00
Lactose	6.00
Soybean oil	2.00
Limestone	0.56
L-Lysine·HCl	0.15
DL-Methionine	0.06
L-Threonine	0.02
Salt	0.40
Vit-mineral, Sow 6^2^	0.30
Total	100.00
Calculated energy and nutrient	
Metabolizable energy, kcal/kg	3487
Net energy, kcal/kg	2615
Crude protein, %	22.97
Ile,^3^%	0.86
Leu,^3^%	1.68
Lys,^3^%	1.35
Met,^3^%	0.44
Thr,^3^%	0.79
Trp,^3^%	0.23
Val,^3^%	0.95
Met + Cys,^3^%	0.74
Ca, %	0.80
Total P, %	0.69
Digestible P, %	0.47
Analyzed nutrients, %	
Dry matter	89.6
Crude protein	22.58
ADF	2.87
NDF	6.99
Ca	1.04
P	0.70

^1^Three additional diets were formulated by adding 10 mg/kg of group A6 type 1-based polymer, 20 mg/kg of group A6 type 1-based polymer (Coligo), or 50 mg/kg of Carbadox to the control diet, respectively

^2^Provided the following quantities of vitamins and micro minerals per kilogram of complete diet: Vitamin A as retinyl acetate, 11,136 IU; vitamin D_3_ as cholecalciferol, 2208 IU; vitamin E as DL-alpha tocopheryl acetate, 66 IU; vitamin K as menadione dimethylprimidinol bisulfite, 1.42 mg; thiamin as thiamine mononitrate, 0.24 mg; riboflavin, 6.59 mg; pyridoxine as pyridoxine hydrochloride, 0.24 mg; vitamin B_12_, 0.03 mg; D-pantothenic acid as D-calcium pantothenate, 23.5 mg; niacin, 44.1 mg; folic acid, 1.59 mg; biotin, 0.44 mg; Cu, 20 mg as copper sulfate and copper chloride; Fe, 126 mg as ferrous sulfate; I, 1.26 mg as ethylenediamine dihydriodide; Mn, 60.2 mg as manganese sulfate; Se, 0.3 mg as sodium selenite and selenium yeast; and Zn, 125.1 mg as zinc sulfate

^3^Amino acids were indicated as standardized ileal digestible AA

After 7 days of adaptation, all pigs were orally inoculated with 3 mL of F18 ETEC for 3 consecutive days from d 0 post-inoculation (PI). The F18 ETEC was originally isolated from a field disease outbreak by the University of Illinois Veterinary Diagnostic Lab (isolate number: U.IL-VDL # 05–27,242). The F18 ETEC expresses heat-labile toxin (LT), heat-stable toxin b (STb), and Shiga-like toxin (Stx2e). The inoculums were prepared by the laboratory of the Western Institute for Food Safety and Security at the University of California, Davis, and were provided at 10^10^ colony-forming unit (CFU) per 3 mL dose in phosphate-buffered saline (PBS). This dose caused mild diarrhea in the current study, consistent with our previous published researches [20–22].

### Clinical observations and sample collections

The procedures for this study were adapted from previous research methods of Liu et al. [20] and Kim et al. [21, 22]. Clinical observations (diarrhea score and alertness score) were recorded twice daily throughout the study. The diarrhea score of each pig was assessed each day visually by two independent evaluators, with the score ranging from 1 to 5 (1 = normal feces, 2 = moist feces, 3 = mild diarrhea, 4 = severe diarrhea, and 5 = watery diarrhea). The frequency of diarrhea was calculated as the percentage of the counting pig days with a diarrhea score 4 or greater. The alertness score of each pig was assessed visually with a score from 1 to 3 (1 = normal, 2 = slightly depressed or listless, and 3 = severely depressed or recumbent). All pigs had an alertness score 1 throughout the study, therefore, data are not reported.

Pigs were weighed on weaning day (d − 7), d 0 before inoculation, d 5, and 11 PI. Feed intake was recorded throughout the study. Average daily gain (ADG), average daily feed intake (ADFI), and feed efficiency (gain:feed) was calculated for each interval from d − 7 to 0, d 0 to 5 PI, and d 5 to 11 PI. Fecal samples were collected from the rectum of all pigs throughout the experiments using a fecal loop or cotton swap on d 2, 5, 8, and 11 PI to test for β-hemolytic coliforms and percentage [20–22]. Twenty-four pigs (3 barrows and 3 gilts from each treatment) were euthanized on d 5 PI near the peak of infection, and the remaining pigs were euthanized at the end of the experiment (d 11 PI) that was the recovery period of the infection. The selection of necropsy time was based on the results of clinical observations and immune response parameters that were reported in previously published research using the same ETEC strain and inoculation dose [21, 22].

To achieve proper restraint and positioning for blood sample collection, pig was placed on a V-shaped table restrained in dorsal recumbence. Blood samples were collected from the jugular vein of all pigs with or without ethylenediaminetetraacetic acid (EDTA) to yield whole blood and serum, respectively, before ETEC challenge (d 0), and on d 2, 5, and 11 PI. Serum samples were collected and immediately stored at − 80 °C before further analysis. Before euthanasia, pigs were anesthetized with a 1-mL mixture of 100 mg telazol, 50 mg ketamine, and 50 mg xylazine (2:1:1) by intramuscular injection. After anesthesia, intracardiac injection with 78 mg sodium pentobarbital (Vortech Pharmaceuticals, Ltd., Dearborn, MI, USA) per 1 kg of BW was used to euthanize each pig. Three 3-cm segments from the duodenum, the middle of the jejunum, and the ileum (10 cm close to the ileocecal junction) were collected and fixed in Carnoy’s solution (ethanol, chloroform, and glacial acetic acid, 6:3:1 v/v/v) for intestinal morphology analysis. Mesenteric lymph nodes were aseptically collected and then pooled within the pig, grounded, diluted, and plated on brain heart infusion agar for measurement of total bacteria, and the results were expressed as CFU per g of lymph node [23, 24]. Spleen samples were analyzed in the same method as mesenteric lymph nodes for bacterial translocation.

### Detection of β-hemolytic coliforms

Briefly, fecal samples were plated on Columbia Blood Agar with 5% sheep blood to identify hemolytic coliforms, which can lyse red blood cells surrounding the colony. Fecal samples were also plated on MacConkey agar to enumerate total coliforms. Hemolytic colonies from the blood agar were sub-cultured on MacConkey agar to confirm that they were lactose-fermenting bacteria and flat pink colonies. All plates were incubated at 37 °C for 24 h in an air incubator. Populations of both total coliforms and β-hemolytic coliforms on blood agar were assessed visually, with a score from 0 to 8 (0 = no bacterial growth, 8 = very heavy bacterial growth). The ratio of scores of β-hemolytic coliforms to total coliforms was calculated. Questionable colonies were sub-sub-cultured on new MacConkey and blood agar plates to verify if they were β-hemolytic *E. coli* by using triple sugar iron agar and lysine iron agar, then verify if they were F18 positive *E. coli* using PCR [25].

### Complete blood count

Whole blood samples were used for measuring total and differential blood cell counts by the Comparative Pathology Laboratory at the University of California, Davis. A multiparameter, automated programmed hematology analyzer (Drew/ERBA Scientific 950 FS Hematological Analyzer, Drew Scientific Inc., Miami, FL) was used for the assay to differentiate porcine blood optimally.

### Measurements of serum cytokine and acute-phase proteins

Serum samples were analyzed for a pro-inflammatory cytokine (Tumor necrosis factor-α; TNF-α; R&D System Inc., Minneapolis, MN, USA) and acute-phase proteins (C-reactive protein and haptoglobin; GenWay Biotech Inc., San Diego, CA, USA) using porcine-specific enzyme-linked immunosorbent assay (ELISA) kits. All samples were analyzed in duplicate, including standard and control. The intra-assay coefficients of variation for TNF-α, C-reactive protein, and haptoglobin were 6.2%, 4.1%, and 2.7%, respectively. The inter-assay coefficients of variation for TNF-α, C-reactive protein, and haptoglobin were 10.0%, 5.6%, and 6.2%, respectively. The results of TNF-α, C-reactive protein, and haptoglobin were expressed in picograms, micrograms, or milligrams per milliliter based on the standard curves.

### Intestinal morphology

The fixed intestinal tissues were embedded in paraffin, sectioned at 5 μm, and stained with high iron diamine and alcian blue. The slides were photographed by an Olympus BX51 microscope at 100 × amplification, and all measurements were conducted in the image processing and analysis software (Image J, NIH). Fifteen straight and integrated villi and their associated crypts and surrounded area were selected to analyze villi height, crypt depth, the number of goblet cells per villus, and cross-sectional area of sulfo- and sialomucin as described by Deplancke and Gaskins [26], and Kim et al. [22].

### Intestinal barrier and innate immunity

Jejunal and ileal mucosa samples were analyzed for gene expression by quantitative real-time PCR (qRT-PCR). Briefly, approximately 100 mg of mucosa sample was homogenized using TRIzol reagent (Invitrogen; Thermo Fisher Scientific, Inc., Waltham, MA, USA). Then total RNA was extracted following RNA extraction procedural guidelines provided by the reagent manufacturer. The RNA quality and quantity were assessed by Agilent Bioanalyzer 2100 (Agilent, Santa Clara, CA, USA). The cDNA was produced from 1 μg of total RNA per sample using the High-Capacity cDNA Reverse Transcription Kit (Applied Biosystems; Thermo Fisher Scientific, Inc., Waltham, MA, USA) in a total volume of 20 μL. The mRNA expression of Claudin 1 (*CLDN1*), Mucin 2 (*MUC2*), Occludin (*OCLN*), and Zonula occludens-1 (*ZO-1*) in jejunal mucosa and the mRNA expression of Interleukin 1 beta (*IL1B*), Interleukin 6 (*IL6*), Cyclooxygenase 2 (*PTGS2*), and Tumor necrosis factor-alpha (*TNF*) in ileal mucosa were analyzed by qRT-PCR. Data normalization was accomplished using beta-actin (*ACTB*) and ribosomal protein L4 (*RPL4*) as housekeeping genes. Primers were designed based on published literature and commercially synthesized by Integrated DNA Technologies, Coralville, IA. All primers were verified prior to qRT-PCR (Table S1). The qRT-PCR reaction conditions followed the published research [27]. The 2^-ΔΔCT^ method was used to analyze the relative expression of genes compared with control [28].

### Statistical analysis

The normality of data was verified with the Shapiro-Wilk test, and outliers were identified using the UNIVARIATE procedure (SAS Inst. Inc., Cary, NC, USA). All data were analyzed by ANOVA using the PROC MIXED of SAS (SAS Institute Inc., Cary, NC, USA) in a randomized complete block design with the pig as the experimental unit. The statistical model included independent variables treatment group, sampling day, and interactions as the fixed effect and blocks as random effects. Treatment means were separated by using the LSMEANS statement and the PDIFF option of PROC MIXED. Contrast statements were used to analyze the dose effects of Coligo. The Chi-square test was used for analyzing the frequency of diarrhea. Statistical significance and tendency were considered at *P* < 0.05 and 0.05 ≤ *P* < 0.10, respectively.

## Results

### Growth performance, diarrhea score, β-hemolytic coliforms

No difference was observed in the initial BW and d 0 BW of pigs among dietary treatments (Table 2). Pigs supplemented with CAR had greater (*P* < 0.05) BW on d 5 PI than pigs in the control and HIGH groups. Pigs supplemented with LOW had the greatest (*P* < 0.05) BW, but pigs supplemented with HIGH had the lowest (*P* < 0.05) BW on d 11 PI among all dietary treatments. Supplementation of LOW had greater (*P* < 0.05) ADFI of pigs from d 5 to 11 PI, compared with control and HIGH groups. Supplementation of Coligo had greater (*P* < 0.05) feed efficiency from d 0 to 5 PI compared with pigs in the control group regardless of dose. Supplementation of HIGH also had greater (*P* < 0.05) feed efficiency of weaned pigs from d 5 to 11 PI, compared with pigs in the control. Pigs fed with CAR had better (*P* < 0.05) feed efficiency than pigs fed with the control diet from d 0 to 5 PI, but this was not the case from d 5 to 11 PI.

**Table 2:** Growth performance of ETEC-infected pigs fed diets supplemented with oligosaccharide-based polymer (Coligo) or antibiotics

	Diet					*P*-value	
Item^1^	Control	LOW^2^	HIGH^3^	CAR^4^	SEM	Diet	Linear^5^
BW, kg							
d − 7	7.21	7.24	7.15	7.34	0.36	0.99	0.90
d 0	8.93	9.27	8.69	9.03	0.36	0.42	0.47
d 5 PI	10.89^b^	11.57^ab^	10.97^b^	11.96^a^	0.35	< 0.05	0.85
d 11 PI*	15.14^ab^	16.58^a^	14.76^b^	16.02^ab^	0.75	< 0.05	0.55
ADG, g							
d − 7 to 0	235	296	211	260	55.3	0.29	0.57
d 0 to 5 PI	393	453	456	533	50.6	0.29	0.35
d 5 to 11 PI*	622	727	705	690	32.9	0.25	0.13
ADFI, g							
d − 7 to 0	424	404	377	331	45.9	0.30	0.35
d 0 to 5 PI	635	631	597	677	34.3	0.49	0.44
d 5 to 11 PI*	803^b^	930^a^	806^b^	893^ab^	57.3	< 0.05	0.94
G:F							
d − 7 to 0	0.59	0.77	0.59	0.71	0.101	0.43	0.97
d 0 to 5 PI	0.57^b^	0.76^a^	0.75^a^	0.77^a^	0.054	< 0.05	< 0.05
d 5 to 11 PI*	0.72^b^	0.79^ab^	0.85^a^	0.83^ab^	0.043	0.07	< 0.05

^a,b^Within a row, means without a common superscript differ (*P* < 0.05)

^1^*BW* body weight, *ADG* average daily gain, *ADFI* average daily feed intake, *G:F* gain:feed, and *PI* post-inoculation. Each least squares mean represents 12 observations, except the *, which has 6 observations

^2^*LOW* Low dose blood group A6 type 1-based polymer (Coligo)

^3^*HIGH* High dose blood group A6 type 1-based polymer (Coligo)

^4^*CAR* carbadox

^5^Linear effects of adding Coligo to the control diet

**Fig. 1:**
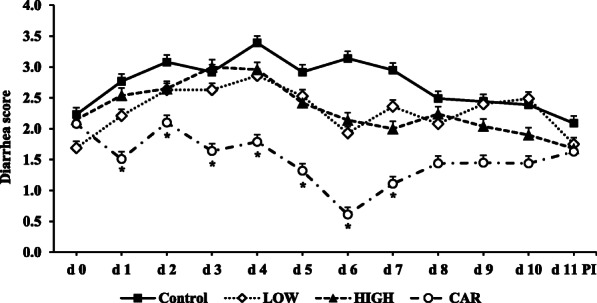
Daily diarrhea score of ETEC-infected weaned pigs fed diets supplemented with oligosaccharide-based polymer (Coligo) or antibiotics. Diarrhea score = 1, normal feces, 2, moist feces, 3, mild diarrhea, 4, severe diarrhea, 5, watery diarrhea. Each least squares mean from d 0 to d 5 post-inoculation (PI) represents 12 observations. Each least squares mean from d 6 to d 11 PI represents 6 observations. *Significant differences were observed among dietary treatment: *P* < 0.05. LOW = Low dose blood group A6 type 1-based polymer (Coligo); HIGH = High dose blood group A6 type 1-based polymer (Coligo); CAR = Carbadox

Pigs supplemented with CAR had the lowest (*P* < 0.05) average diarrhea score from d 0 to 5 PI and d 5 to 11 PI among all dietary treatments (Table 3; Fig. 1). Compared with pigs in control group, pigs supplemented with LOW had lower (*P* < 0.05) average diarrhea score of weaned pigs from d 0 to 5 PI, but this was not the case from d 5 to 11 PI. Supplementation of CAR or any dose of Coligo had lower (*P* < 0.05) frequency of diarrhea of weaned pigs from d 0 to 11 PI.

**Table 3:** Diarrhea score and frequency of diarrhea of ETEC-infected weaned pigs fed diets supplemented with oligosaccharide-based polymer (Coligo) or antibiotics

Diarrhea score	Diet				*P*-value		
	Control	LOW^1^	HIGH^2^	CAR^3^	SEM	Diet	Linear^4^
d 0–5^5^	2.88^a^	2.38^b^	2.62^ab^	1.78^c^	0.17	< 0.01	0.15
d 5–11^6^	2.60^a^	2.17^a^	2.01^a^	1.28^b^	0.31	< 0.01	0.06
Pig days	120	109	120	105			
Frequency of diarrhea^7^	27.50^a^	13.76^b^	14.17^b^	7.62^b^	–	< 0.01	–

^a,b,c^Within a row, means without a common superscript differ (*P* < 0.05)

^1^*LOW* Low dose blood group A6 type 1-based polymer (Coligo)

^2^*HIGH* High dose blood group A6 type 1-based polymer (Coligo)

^3^*CAR* carbadox

^4^Linear effects of adding Coligo to the control diet

^5^Each least squares mean represents 12 observations

^6^Each least squares mean represents 6 observations

^7^Frequency = number of pen days with fecal score ≥ 4

**Fig. 2:**
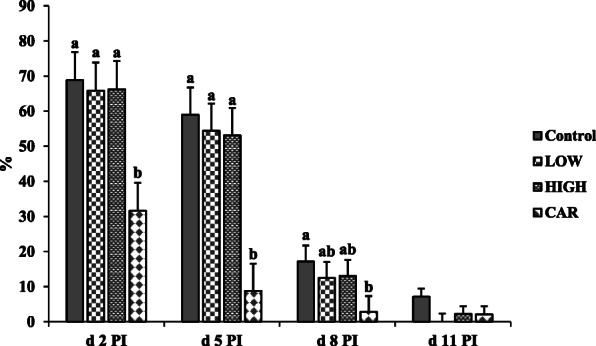
The percentage (%) of β-hemolytic coliform in fecal samples of ETEC-infected pigs fed diets supplemented with oligosaccharide-based polymer (Coligo) or antibiotics. Each least squares mean from d 0 to d 5 post-inoculation (PI) represents 12 observations. Each least squares mean from d 6 to d 11 PI represents 6 observations. ^a,b^Means without a common superscript differ (*P* < 0.05). LOW = Low dose blood group A6 type 1-based polymer (Coligo); HIGH = High dose blood group A6 type 1-based polymer (Coligo); CAR = Carbadox

No β-hemolytic coliform was observed in the feces of all pigs before ETEC inoculation. Pigs supplemented with CAR had the lowest (*P* < 0.05) β-hemolytic coliform percentage in feces on d 2 and 5 PI among all dietary treatments (Fig. 2). The percentage of β-hemolytic coliform in feces was not different between Coligo groups and CAR on d 8 PI. There were no differences observed in fecal culture on d 11 PI among the treatments.

### Systemic immunity and red blood cell profile

Lymphocyte counts were greater (*P* < 0.05) in pigs fed CAR on d 0 before ETEC inoculation (Table 4). Pigs in the LOW group had lower (*P* < 0.05) neutrophils, lymphocytes, and basophils on d 2 PI and lower (*P* < 0.05) neutrophil counts on d 5 PI, compared with pigs in the control group. Supplementation of HIGH also had lower (*P* < 0.05) white blood cell counts, neutrophils, lymphocytes, and basophils on d 2 PI. Pigs in the CAR group had lower (*P* < 0.05) neutrophils and basophils on d 2 PI and lower (*P* < 0.05) neutrophils on d 5 PI, but higher (*P* < 0.05) eosinophils on d 5 PI, compared with pigs in control group. No difference was observed in white blood cell profiles among treatments on d 11 PI.

**Table 4:** Total and differential white blood cells, and serum cytokine and acute-phase proteins in ETEC-infected weaned pigs fed diets supplemented with oligosaccharide-based polymer (Coligo) or antibiotics

	Diet					*P*-value	
Item^1^	Control	LOW^2^	HIGH^3^	CAR^4^	SEM	Diet	Linear5
d 0 before infection							
WBC, 10^3^/μL	15.28	16.91	15.70	16.85	1.00	0.58	0.77
Neu, 10^3^/μL	7.93	9.03	8.43	7.56	0.67	0.46	0.60
Lym, 10^3^/μL	6.39^b^	6.72^b^	6.08^b^	8.26^a^	0.48	< 0.05	0.65
Mono, 10^3^/μL	0.66	0.83	0.79	0.75	0.11	0.73	0.41
Eos, 10^3^/μL	0.24	0.27	0.35	0.20	0.10	0.64	0.33
Baso, 10^3^/μL	0.057	0.072	0.051	0.075	0.018	0.074	0.81
Serum							
TNF-α, pg/mL	63.56	59.67	61.51	72.80	1.62	0.98	0.99
C-reactive protein, μg/mL	7.37	6.86	8.57	8.08	1.39	0.86	0.46
Haptoglobin, g/mL	0.984	0.730	1.211	1.072	0.135	0.065	0.031
d 2 PI							
WBC, 10^3^/μL	21.16^a^	18.04^ab^	17.78^b^	18.53^ab^	1.01	< 0.05	< 0.05
Neu, 10^3^/μL	12.25^a^	9.83^b^	9.79^b^	8.34^b^	0.96	< 0.05	< 0.05
Lym, 10^3^/μL	7.27^ab^	6.97^b^	6.79^b^	8.54^a^	0.41	< 0.05	0.41
Mono, 10^3^/μL	1.16	1.05	0.85	1.33	0.16	0.29	0.21
Eos, 10^3^/μL	0.40	0.25	0.19	0.23	0.10	0.09	< 0.05
Baso, 10^3^/μL	0.106^a^	0.036^b^	0.039^b^	0.029^b^	0.017	< 0.05	< 0.05
Serum							
TNF-α, pg/mL	107.92	67.06	66.28	66.28	32.89	0.63	0.28
C-reactive protein, μg/mL	20.42^a^	22.74^a^	22.88^a^	12.38^b^	2.693	0.018	0.65
Haptoglobin, g/mL	1.306^ab^	1.142^b^	1.561^a^	1.066^b^	0.127	< 0.05	0.11
d 5 PI							
WBC, 10^3^/μL	21.00	18.56	18.67	18.19	1.09	0.14	0.15
Neu, 10^3^/μL	10.93^a^	9.17^b^	9.54^ab^	7.89^b^	0.54	< 0.05	< 0.05
Lym, 10^3^/μL	9.03	8.22	8.02	8.35	0.65	0.69	0.25
Mono, 10^3^/μL	0.86	0.86	0.79	1.21	0.15	0.29	0.74
Eos, 10^3^/μL	0.13^b^	0.26^ab^	0.25^ab^	0.55^a^	0.14	< 0.05	0.41
Baso, 10^3^/μL	0.059	0.043	0.078	0.057	0.016	0.49	0.41
Serum							
TNF-α, pg/mL	90.64	68.36	54.39	32.99	32.88	0.25	0.16
C-reactive protein, μg/mL	21.61^a^	17.46^ab^	19.84^ab^	15.96^b^	1.72	0.082	0.93
Haptoglobin, g/mL	1.655^a^	1.084^b^	1.348^ab^	1.154^b^	0.128	0.081	0.93
d 11 PI							
WBC, 10^3^/μL	16.15	17.40	17.32	14.92	1.58	0.64	0.57
Neu, 10^3^/μL	8.68	10.71	9.67	8.12	1.22	0.16	0.40
Lym, 10^3^/μL	6.33	6.69	7.21	6.39	0.63	0.76	0.35
Mono, 10^3^/μL	0.66	1.12	0.99	1.39	0.19	0.10	0.21
Eos, 10^3^/μL	0.18	0.31	0.24	0.21	0.13	0.74	0.57
Baso, 10^3^/μL	0.058	0.083	0.048	0.042	0.025	0.51	0.70
Serum							
TNF-α, pg/mL	82.58	84.62	72.71	59.39	28.03	0.96	0.79
C-reactive protein, μg/mL	21.95^a^	17.71^ab^	18.29^ab^	12.61^b^	2.89	0.091	0.65
Haptoglobin, g/mL	0.819	0.599	0.538	0.412	0.178	0.24	0.31

^a,b^Within a row, means without a common superscript differ (*P* < 0.05)

^1^*WBC* white blood cell, *Neu* neutrophil, *Lym* lymphocyte, *Mono* monocyte, *Eos* eosinophil, *Baso* basophil, *PI* post-inoculation. Each least squares mean represents 12 observations, except d 11 PI that has 6 observations

^2^*LOW* Low dose blood group A6 type 1-based polymer (Coligo)

^3^*HIGH* High dose blood group A6 type 1-based polymer (Coligo)

^4^*CAR* carbadox

^5^Linear effects of adding Coligo to the control diet

No difference was observed in serum TNF-α concentration among dietary treatments throughout the experiment. Compared with the pigs fed control diet, pigs supplemented with LOW had lower (*P* < 0.05) haptoglobin on d 5 PI, while pigs fed CAR had lower (*P* < 0.05) C-reactive protein on d 2, 5, and 11 PI and had lower (*P* < 0.05) haptoglobin on d 5 PI. No differences in serum C-reactive protein and haptoglobin were observed between the control and HIGH groups.

Before ETEC inoculation, pigs in the CAR group had the lowest (*P* < 0.05) mean corpuscular volume and total platelets among all dietary treatments on d 0 (Table S2). Supplementation of LOW had lower (*P* < 0.05) red blood cells and packed cell volume on d 2 PI, while supplementation of HIGH had lower (*P* < 0.05) packed cell volume on d 5 PI, compared with pigs in the control. Pigs supplemented with CAR had lower (*P* < 0.05) red blood cells and packed cell volume, but higher (*P* < 0.05) mean corpuscular hemoglobin and mean corpuscular hemoglobin concentration on d 2 and 5 PI, compared with pigs in the control. Supplementation of CAR also had greater (*P* < 0.05) total protein concentration on d 11 PI in comparison to pigs in the other treatments.

### Bacterial translocation

**Fig. 3:**
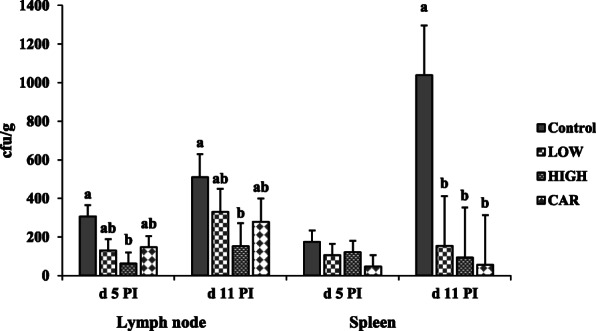
Bacterial counts (CFU/g) in lymph node and spleen of ETEC-infected weaned pigs fed diets supplemented with oligosaccharide-based polymer (Coligo) or antibiotics. Each least squares mean from d 0 to d 5 post-inoculation (PI) represents 12 observations. Each least squares mean from d 6 to d 11 PI represents 6 observations. ^a,b^Means without a common superscript differ (*P* < 0.05). LOW = Low dose blood group A6 type 1-based polymer (Coligo); HIGH = High dose blood group A6 type 1-based polymer (Coligo); CAR = Carbadox

Supplementation of HIGH had lower (*P* < 0.05) bacterial translocation in lymph nodes on d 5 and 11 PI compared with control group (Fig. 3). Pigs supplemented with Coligo or CAR had lower (*P* < 0.05) bacterial translocation in the spleen than pigs in the control on d 11 PI.

### Intestinal morphology

On d 5 PI, supplementation of Coligo dose-dependently had greater (linear, *P* < 0.05) villi height, the ratio of villi height to crypt depth, villi width, and villi area in duodenum, had greater (linear, *P* < 0.05) the ratio of villi height to crypt depth in jejunum, and had greater (linear, *P* < 0.05) villi height, the ratio of villi height to crypt depth, and villi area in ileum, compared with the control group (Table S3). Supplementation of Coligo also had greater (linear, *P* < 0.05) duodenal and jejunal villi height and jejunal and ileal villi area, and tended to have greater (linear, *P* < 0.10) the ratio of villi height to crypt depth in jejunum and ileal villi height on d 11 PI. Pigs fed with CAR had greater (*P* < 0.05) villi height in duodenum and ileum, the ratio of villi height to crypt depth in all three intestinal segments, and villi area in duodenum than pigs in the control group on d 5 PI. On d 11 PI, pigs supplemented with CAR had higher (*P* < 0.05) villi height in all three intestinal segments, greater (*P* < 0.05) villi height to crypt depth ratio in jejunum, and bigger (*P* < 0.05) sialomucin area in duodenum than pigs in the control group. In addition, pigs in the CAR group also had greater (*P* < 0.05) villi height:crypt depth in all intestinal segments on d 5 PI, and greater (*P* < 0.05) villi height in ileum, in comparison to pigs in the Coligo treatment group.

### Intestinal barrier and innate immunity

**Fig. 4:**
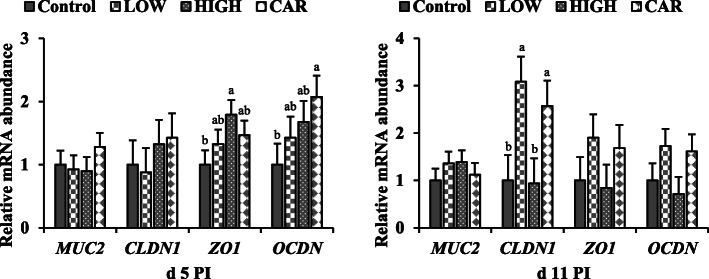
Gene expression profiles in jejunal mucosa of ETEC-infected weaned pigs fed diets supplemented with oligosaccharide-based polymer (Coligo) or antibiotics on d 5 or 11 post-inoculation (PI). ^a,b^Means without a common superscript differ (*P* < 0.05). Each least squares mean represents 6 observations. LOW = Low dose blood group A6 type 1-based polymer (Coligo); HIGH = High dose blood group A6 type 1-based polymer (Coligo); CAR = Carbadox; *MUC2* = Mucin-2; *CLDN1* = Claudin-1; *ZO-1* = Zonula occludens-1; *OCDN* = Occludin

**Fig. 5:**
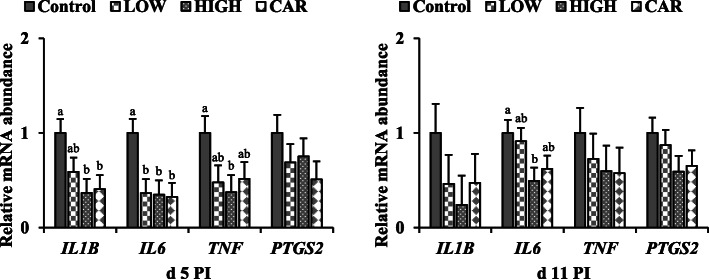
Gene expression profiles in ileal mucosa of ETEC-infected weaned pigs fed diets supplemented with oligosaccharide-based polymer (Coligo) or antibiotics on d 5 or 11 post-inoculation (PI). ^a,b^Means without a common superscript differ (*P* < 0.05). Each least squares mean represents 6 observations. LOW = Low dose blood group A6 type 1-based polymer (Coligo); HIGH = High dose blood group A6 type 1-based polymer (Coligo); CAR = Carbadox; *IL1B*: Interleukin-1 beta; *IL6*: Interleukin-6; *TNF* = Tumor necrosis factor; *PTGS2*: Cyclooxygenase-2

No difference was observed in the mRNA expression of *MUC2* in jejunal mucosa among pigs in all dietary treatment groups (Fig.4). On d 5 PI, supplementation of HIGH up-regulated (*P* < 0.05) the mRNA expression of *ZO1* and addition of CAR had greater (*P* < 0.05) mRNA expression of *OCLN*, compared with pigs in control group. On d 11 PI, supplementation of LOW or CAR had higher (*P* < 0.05) mRNA expression of *CLDN1* in jejunal mucosa of weaned pigs, compared with the control and HIGH groups. On d 5 PI, supplementation of LOW down-regulated (*P* < 0.05) the mRNA expression of *IL6*, supplementation of HIGH had lower (*P* < 0.05) mRNA expression of *IL1B*, *IL6*, and *TNF*, and supplementation of CAR had lower (*P* < 0.05) *IL1B* and *IL6* gene expression in ileal mucosa of weaned pigs in comparison to control pigs (Fig. 5). Supplementation of HIGH also had lower (*P* < 0.05) *IL6* mRNA expression on d 11 PI in ileal mucosa, compared with the control group. However, no differences were observed in the gene expression of inflammatory mediators among LOW, HIGH, and CAR groups.

## Discussion

ETEC infection is initiated by bacterial attachment to specific receptors on the intestinal epithelium by fimbrial adhesins, followed by colonization of ETEC in the small intestine [29]. Once colonization is established, ETEC rapidly proliferate and produce one or more enterotoxins, which can stimulate water and electrolyte secretion and reduce fluid absorption in the small intestine and induce diarrhea [30]. Diarrhea caused by ETEC is one of the most prevalent diseases during the weaning stage, which is responsible for anorexia, slower growth, or even the death of pigs. Results of the present study demonstrated that supplementation of Coligo improved growth rate, and reduced frequency of diarrhea and systemic inflammation of weaned pigs experimentally challenged with F18 ETEC. The potential mechanisms of action include inhibition of binding of bacteria and as such colonization of the gut by the F18 ETEC [11, 13], enhancing gut barrier function and reducing local and systemic inflammation.

In the current study, pigs in the control group grew slower and had a high frequency of diarrhea compared to pigs without ETEC challenge in our previous research [20, 22]. These observations, combined with the presence of β-hemolytic coliforms in feces, confirmed that pigs were successfully infected with F18 ETEC*.* In agreement with our previous research, the peak of F18 ETEC infection was present approximately 5 days post-inoculation, and most pigs moved into the recovery stage on day 11 to 12 post-inoculation. Results of the current study have demonstrated that pigs supplemented with Coligo or antibiotics had reduced frequency of diarrhea and enhanced growth performance than pigs in control group, indicating both supplements could protect pigs against F18 ETEC infection.

In agreement with the diarrhea severity results, pigs supplemented with antibiotics had less β-hemolytic coliforms compared with pigs in the control group, indicating lowered F18 ETEC shedding in pig’s feces during the peak infection period. The exact mechanisms of action of carbadox are not fully understood, but it has been suggested that virulence activity was disabled by interfering with DNA synthesis in Gram-negative bacteria, including *E. coli* [31, 32]. However, pigs supplemented with Coligo had a relatively higher percentage of β-hemolytic coliforms than pigs fed with antibiotics. These observations indicated that the beneficial effects of Coligo and antibiotics on reducing weaned pigs’ diarrhea were through different mechanisms. It has been reported that heat-labile toxin expressed by ETEC binds blood group antigens, with a preference for A-epitope [33]. Moreover, it has been hypothesized that blood group A antigen might disturb the toxin activity by interfering with ETEC binding to the receptors in the small intestine of pigs [34]. Coddens et al. [10] also observed a high correlation between blood group A antigen and F18 ETEC adherence in the small intestine of young pigs in vitro. Moreover, it has been demonstrated that F18 *E. coli* specifically interact with glycosphingolipids possess blood group ABH determinants on a type 1 core, which were identified as the cell surface receptors for F18 fimbrial binding to the small intestinal epithelium [11]. With these specific features, the addition of extra blood group A antigen could enhance the binding affinity of F18 ETEC to polymers. Thus, fecal culture results suggest that the Coligo polymer may reduce the ETEC attachment to the small intestine and accelerate the excretion of these pathogenic bacteria from their gastrointestinal tract. Taken altogether, antibiotics or Coligo may help pigs recover from ETEC infection through different mechanisms, which we attempted to explore in the current research.

Tight junctions play critical roles in maintaining the integrity of intestinal structure and barrier, and regulating intestinal paracellular permeability [35, 36]. Several multi-protein complexes, including zonulae occludens (ZO), occludins, and claudins, are involved in the tight junction barrier. Previous studies have reported that ETEC infection impairs intestinal barrier function by down-regulating tight junction protein expression, leading to intestinal inflammation [22, 37]. Morphological lesions, such as loss of villus absorptive cells, villus atrophy, and intestinal permeability disturbances are also observed in the small intestine of pigs with ETEC infection [1, 38]. In the present study, pigs supplemented with Coligo or antibiotics had greater mRNA expression of *ZO1* or *OCDN* at the peak of ETEC infection, respectively, and greater *CLDN1* expression on d 11 PI than pigs in control group. ZO-1 protein connects and interacts with junctional proteins, such as occludins and claudins, to form the physical barrier, which determines the permselectivity of the paracellular diffusion pathway [39]. The up-regulation of mRNA expression of tight junction proteins in this study suggests the protective effects of Coligo or antibiotics on intestinal barrier function against ETEC infection. Consistently, pigs in Coligo groups or antibiotics group had higher villi height, greater villi height to crypt depth ratio, and villi area, demonstrating a preventive effect against intestinal structure disruption. Overall, these results demonstrated that supplementation of Coligo or antibiotics enhanced gut integrity and morphology of weaned pigs, which was one of the major reasons these pigs grew faster than pigs in control group.

Bacterial translocation is defined as the passage of viable bacteria from the gastrointestinal tract to normally sterile tissues, such as mesenteric lymph nodes and other internal organs, including the spleen [40, 41]. The major mechanisms promoting bacterial translocation are intestinal bacterial overgrowth, deficiencies in host immune defenses by disturbed gut integrity, and increased permeability or mucosal injury [42]. It was previously reported that bacterial translocation to mesenteric lymph nodes was increased in pigs challenged with ETEC [43, 44]. In the current study, pigs supplemented with antibiotics lowered bacterial populations in the spleen, and pigs fed with Coligo had lower bacterial populations in both mesenteric lymph nodes and spleen than pigs in the control group. These observations clearly supported that supplementation of Coligo or antibiotics reduced the damage of ETEC infection on the gut integrity of weaned pigs compared with control pigs. Overall, results of tight junction protein mRNA expression, intestinal morphology, and bacterial translocation imply that pigs in Coligo and antibiotics groups may have better intestinal health, which would be responsible for better nutrient digestion and absorption, and hence better performance.

The colonized F18 ETEC could produce large quantities of toxins, such as heat-labile toxins, heat-stable toxins, Shiga toxins, and lipopolysaccharides [45]. Those toxins induce functional changes in the small intestinal epithelial cells, as well as stimulate the synthesis of cytokines and acute-phase proteins (e. g. C-reactive protein and haptoglobin), followed by systemic and local inflammation [1, 46]. Our previously published research that used the same bacteria strain, F18 ETEC, reported that ETEC infection could induce systemic inflammation, such as increasing white blood cell counts, neutrophils, and lymphocytes, as well as enhancing several pro-inflammatory cytokines and acute-phase protein concentrations in serum of weaned pigs [20, 47]. In the current study, pigs supplemented with antibiotics had reductions in neutrophils and serum concentrations of C-reactive protein and haptoglobin during the peak infection period. Similarly, pigs supplemented with Coligo had lower numbers of white blood cells, neutrophils, and lymphocytes on d 2 PI, and lower neutrophils on d 5 PI. In addition, serum haptoglobin concentrations were also relatively lower in the pigs supplemented with Coligo. These findings demonstrated the capacity of antibiotics and Coligo polymer in alleviating ETEC-induced systemic inflammation, possibly by reducing the bacterial population in pigs’ gut.

In response to an infectious challenge, it is well recognized that innate immune responses have essential roles in preventing and suppressing inflammation [48]. Pathogen recognition receptors initiate the innate immune response on intestinal epithelial cells to detect and recognize the pathogen-associated molecular patterns, such as microbial membranes, resulting in a rapid release of proinflammatory cytokines [49]. It has been reported that secreted proinflammatory cytokines, which are primarily involved in host responses to disease or infection, could induce intestinal inflammation, tissue destruction, and nutrient and ion malabsorption in pigs [50, 51]. Moreover, numerous studies have confirmed the induction of proinflammatory cytokines in porcine intestinal epithelial cells caused by ETEC infection [52–54]. The elevation of proinflammatory cytokines by ETEC challenge has nutrient cost, thus also contributing to the reduced growth performance of pigs [55]. In the current study, mRNA expression of proinflammatory markers (i.e., *IL1B, IL6,* and *TNF*) in ileal mucosa were down-regulated by Coligo supplementation, which is consistent with the results of systemic immunity. These observations can be accounted for by assuming not only reduced the attachment of ETEC to the intestinal epithelium by blood group oligosaccharides, but also potentially modified intestinal microbiota by Coligo. It was reported that porcine blood type AO could be a possible factor influencing microbiota composition [56] and Priori et al. [57] suggest changes in the intestinal microbiota are affected by porcine blood group. Thus, the blood group A antigen in Coligo may affect the composition and function of microbial communities when fed to pigs. Moreover, recent studies demonstrated that dietary supplementation of ε-PL altered ileal microbiota structure and function in pigs [58] and fecal microbial community in mice [59]. ε-PL supplementation may promote the growth of beneficial microorganisms in the intestinal tract, therefore, reducing the proliferation of pathogens. The exact mechanisms of ε-PL in the current study remain unclear, so further research is needed to confirm the effects of Coligo on the pigs’ gut microbial community, intestinal inflammation, and immune responses against F18 ETEC. Pigs supplemented antibiotics also had reductions in mRNA expression of proinflammatory markers in the present study. This finding demonstrated that pigs supplemented antibiotics has less severe intestinal inflammation than pigs in control group. In agreement with previous research, antibiotic supplements might exert anti-inflammatory properties in the intestine or accumulate in phagocytic inflammatory cells, therefore, attenuating inflammatory responses in animals [60, 61]. Taken altogether, down-regulation in mRNA expression of proinflammatory cytokines by Coligo or antibiotics supplementation is beneficial for pigs in terms of their intestinal health and growth performance.

In conclusion, results in the current study suggest that in-feed supplementation of Coligo or antibiotic (carbadox) enhanced growth performance and reduced the severity of diarrhea caused by ETEC F18 infection. Although the percentage of β-hemolytic coliforms in fecal samples of pigs fed with Coligo was less diminished than pigs supplemented with antibiotics, enhanced disease resistance was demonstrated by the improved gut barrier integrity and attenuated systemic and intestinal inflammation. To further explore the mechanisms of action of Coligo, integrated metabolomics and metagenomics approaches may be considered to provide more insights into the beneficial effects of Coligo or other polymers on pigs’ health. Overall, the current study indicates that supplementation with Coligo has promising impacts on promoting growth and disease resistance of newly weaned pigs infected with ETEC F18. The efficacy of Coligo is comparable to antibiotic (carbadox) demonstrating the potential of Coligo as antibiotic alternative for animal growth performance and disease resistance. Large-scale animal trials are recommended to further evaluate the impacts of Coligo on performance of weaned pigs under commercial practice conditions.

## Supplementary Information

**Additional file 1.** Table S1 Gene-specific primer sequences and PCR conditions.

**Additional file 2.** Table S2 Red blood cell profiles of ETEC-infected weaned pigs fed diets supplemented with oligosaccharide-based polymer (Coligo) or antibiotics.

**Additional file 3.** Table S3 Intestinal morphology of ETEC-infected weaned pigs fed diets supplemented with oligosaccharide-based polymer (Coligo) or antibiotics.

## Data Availability

All data generated or analyzed during this study are available from the corresponding author upon reasonable request.

## Abbreviations

ADFI: Average daily feed intake
ADG: Average daily gain
BW: Body weight
CFU: Colony-forming unit
*CLDN1*: Claudin-1
ETEC: Enterotoxigenic *E. coli*
*IL1B*: Interleukin-1 beta
*IL6*: Interleukin-6
mRNA: Messenger RNA
*MUC2*: Mucin-2
*OCDN*: Occludin
PI: Post-inoculation
qRT-PCR: Quantitative real-time polymerase chain reaction
*TNF*: Tumor necrosis factor
*ZO-1*: Zona occludens-1
