# Supplementary material for: Supplementation of oligosaccharide-based polymer enhanced growth and disease resistance of weaned pigs by modulating intestinal integrity and systemic immunity
Source: J Anim Sci Biotechnol. 2022 Jan 12;13:10. doi: 10.1186/s40104-021-00655-2 (PMC8753815; doi:10.1186/s40104-021-00655-2)
Supplement: Supplementary file 1 — Additional file 1. Table S1 Gene-specific primer sequences and PCR conditions. [file 40104_2021_655_MOESM1_ESM.docx]

**Table S1.** Gene-specific primer sequences and PCR conditions**^1^**

| Gene^2^ | Acc. No^3^ | Forward primer (5’-3’) | Reverse primer (5’-3’) |
| --- | --- | --- | --- |
| *MUC2* | AK231524 | CAACGGCCTCTCCTTCTCTGT | GCCACACTGGCCCTTTGT |
| *CLDN1* | NM001244539 | TCTTAGTTGCCACAGCATGG | CCAGTGAAGAGAGCCTGACC |
| *ZO-1* | AJ318101 | CCGCCTCCTGAGTTTGATAG | CAGCTTTAGGCACTGTGCTG |
| *OCDN* | NM001163647 | CAGCCAACGGGAAGATTCTG | ATGGCTTCCAGGTCGTCAT |
| *IL1B* | NM 214055.1 | CCTTGAAACGTGCAATGATG | TTCAAGTCCCCTGTGAGGAG |
| *IL6* | AB194100.1 | CTGGCAGAAAACAACCTGAACC | TGATTCTCATCAAGCAGGTCTCC |
| *TNFA* | EU682384.1 | AACCTCAGATAAGCCCGTCG | ACCACCAGCTGGTTGTCTTT |
| *PTGS2* | [AF207824.1](https://www.ncbi.nlm.nih.gov/nucleotide/AF207824.1?report=genbank&log$=nucltop&blast_rank=3&RID=4RV9ZEVM014) | ATAAGTGTGACTGCACCCGAAC | GGTGGGCTATCAATCAGATGTG |
| *ACTB* | DQ452569 | TTCCAGCAGATGTGGATCAG | CATGCCAATCTCATCTC |
| *RPL4* | XM_003121741 | CAAGAGTAACTACAACCTTC | GAACTCTACGATGAATCTTC |

^1^Thermal cycling conditions were 95°C for 20 sec and 95°C for 1 sec, followed by 40 cycles with 20 sec at 60°C.

^2^ *MUC2*: Mucin-2; *CLDN1* = Claudin-1; *ZO-1*: Zonula occludens-1; *OCDN* = Occludin; *IL1B*: Interleukin-1 beta; *IL6*: Interleukin-6; *TNFA* = Tumor necrosis factor-alpha; *PTGS2*: Cyclooxygenase-2; *ACTB*: Beta-actin; *RPL4*: Ribosomal protein L4.

^3^Accession number in GenBank database.
