# Supplementary material for: Supplementation of oligosaccharide-based polymer enhanced growth and disease resistance of weaned pigs by modulating intestinal integrity and systemic immunity
Source: J Anim Sci Biotechnol. 2022 Jan 12;13:10. doi: 10.1186/s40104-021-00655-2 (PMC8753815; doi:10.1186/s40104-021-00655-2)
Supplement: Supplementary file 2 — Additional file 2. Table S2 Red blood cell profiles of ETEC-infected weaned pigs fed diets supplemented with oligosaccharide-based polymer (Coligo) or antibiotics. [file 40104_2021_655_MOESM2_ESM.docx]

**Table S2.** Red blood cell profiles of ETEC-infected weaned pigs fed diets supplemented with oligosaccharide-based polymer (Coligo) or antibiotics

|  | Diet | | | | |  | | | | | | *P*-value | | | | |
| --- | --- | --- | --- | --- | --- | --- | --- | --- | --- | --- | --- | --- | --- | --- | --- | --- |
| Item^1^ | Control | | LOW^2^ | | HIGH^3^ | | | CAR^4^ | | SEM | | | Diet | | Linear^5^ | |
| d 0 before infection |  | |  | |  | | |  | |  | | |  | |  | |
| RBC, 10^6^/μL | 7.16 | | 6.68 | | 7.24 | | | 7.21 | | 0.31 | | | 0.47 | | 0.83 | |
| HGB, g/dL | 9.80 | | 9.75 | | 10.22 | | | 10.11 | | 0.37 | | | 0.77 | | 0.43 | |
| HCT, % | 31.63 | | 30.58 | | 31.88 | | | 30.59 | | 1.13 | | | 0.78 | | 0.88 | |
| MCV, fL^6^ | 44.68^ab^ | | 46.15^a^ | | 44.51^ab^ | | | 42.16^b^ | | 1.27 | | | <0.05 | | 0.90 | |
| MCH, pg | 13.88 | | 14.72 | | 14.28 | | | 14.04 | | 0.43 | | | 0.41 | | 0.45 | |
| MCHC, g/dL | 31.02^b^ | | 31.98^ab^ | | 32.04^ab^ | | | 33.10^a^ | | 0.47 | | | <0.05 | | 0.10 | |
| RDW, % | 26.59 | | 26.38 | | 25.82 | | | 25.49 | | 0.86 | | | 0.71 | | 0.46 | |
| Platelets, 10^3^/μL | 420^a^ | | 390^a^ | | 453^a^ | | | 291^b^ | | 46.2 | | | <0.01 | | 0.45 | |
| MPV, fL^5^ | 14.38 | | 9.27 | | 9.34 | | | 9.80 | | 2.59 | | | 0.42 | | 0.16 | |
| Total protein,  g/dL | 4.70 | | 4.69 | | 4.82 | | | 4.99 | | 0.11 | | | 0.16 | | 0.42 | |
| d 2 PI |  | |  | |  | | |  | |  | | |  | |  | |
| RBC, 10^6^/μL | 6.79^a^ | | 6.06^b^ | | 6.53^ab^ | | | 6.01^b^ | | 0.20 | | | <0.05 | | 0.45 | |
| HGB, g/dL | 9.48 | | 9.07 | | 9.39 | | | 9.30 | | 0.27 | | | 0.68 | | 0.79 | |
| HCT, % | 30.26^a^ | | 28.13^b^ | | 28.76^ab^ | | | 25.91^c^ | | 0.73 | | | <0.05 | | 0.12 | |
| MCV, fL^5^ | 45.21^ab^ | | 47.36^a^ | | 44.60^ab^ | | | 42.93^b^ | | 1.38 | | | <0.05 | | 0.69 | |
| MCH, pg | 14.14^b^ | | 15.09^ab^ | | 14.43^b^ | | | 15.54^a^ | | 0.34 | | | <0.05 | | 0.55 | |
| MCHC, g/dL | 31.50^b^ | | 32.05^b^ | | 32.63^b^ | | | 36.00^a^ | | 0.80 | | | <0.01 | | 0.22 | |
| RDW, % | 26.75 | | 26.87 | | 26.57 | | | 27.08 | | 1.26 | | | 0.99 | | 0.91 | |
| Platelets, 10^3^/μL | 384 | | 423 | | 392 | | | 478 | | 40.1 | | | 0.39 | | 0.88 | |
| MPV, fL^5^ | 9.30 | | 9.64 | | 9.91 | | | 9.93 | | 0.38 | | | 0.56 | | 0.23 | |
| Total protein,  g/dL | 4.71 | | 4.53 | | 4.70 | | | 4.76 | | 0.15 | | | 0.69 | | 0.96 | |
| d 5 PI |  | |  | |  | | |  | |  | | |  | |  | |
| RBC, 10^6^/μL | 6.53^a^ | | 6.42^ab^ | | 6.05^ab^ | | | 5.90^b^ | | 0.18 | | | <0.05 | | 0.07 | |
| HGB, g/dL | 9.14^ab^ | | 9.57^a^ | | 8.54^b^ | | | 9.54^a^ | | 0.25 | | | <0.05 | | 0.11 | |
| HCT, % | 30.02^a^ | | 30.47^a^ | | 27.15^b^ | | | 26.76^b^ | | 0.89 | | | <0.05 | | <0.05 | |
| MCV, fL^5^ | 46.08^ab^ | | 47.72^a^ | | 45.24^b^ | | | 44.32^b^ | | 1.19 | | | <0.05 | | 0.47 | |
| MCH, pg | 14.07^c^ | | 14.95^b^ | | 14.13^c^ | | | 16.09^a^ | | 0.23 | | | <0.01 | | 0.85 | |
| MCHC, g/dL | 30.63^b^ | | 31.46^b^ | | 31.55^b^ | | | 36.34^a^ | | 0.82 | | | <0.01 | | 0.22 | |
| RDW, % | 28.06^ab^ | | 28.58^ab^ | | 26.73^b^ | | | 30.36^a^ | | 1.22 | | | <0.05 | | 0.26 | |
| Platelets, 10^3^/μL | 521 | | 485 | | 514 | | | 530 | | 34.7 | | | 0.83 | | 0.88 | |
| MPV, fL^5^ | 9.43 | | 9.85 | | 9.85 | | | 9.57 | | 0.27 | | | 0.59 | | 0.26 | |
| Total protein,  g/dL | 4.59 | | 4.54 | | 4.59 | | | 4.66 | | 0.11 | | | 0.91 | | 0.96 | |
| d 11 PI |  |  | |  | | |  | |  | |  | | |  | |  |
| RBC, 10^6^/μL | 6.34 | | 6.55 | | 6.29 | | | 6.28 | | 0.18 | | | 0.52 | | 0.77 | |
| HGB, g/dL | 10.06 | | 10.37 | | 9.60 | | | 10.34 | | 0.37 | | | 0.14 | | 0.18 | |
| HCT, % | 30.76 | | 31.61 | | 29.45 | | | 28.47 | | 1.28 | | | 0.10 | | 0.24 | |
| MCV, fL^5^ | 48.31 | | 48.32 | | 47.23 | | | 45.19 | | 1.29 | | | 0.26 | | 0.49 | |
| MCH, pg | 15.90 | | 15.80 | | 15.29 | | | 16.42 | | 0.26 | | | 0.09 | | 0.13 | |
| MCHC, g/dL | 33.02^b^ | | 32.76^b^ | | 32.52^b^ | | | 36.39^a^ | | 0.82 | | | <0.05 | | 0.64 | |
| RDW, % | 26.65 | | 26.19 | | 26.78 | | | 28.40 | | 1.40 | | | 0.69 | | 0.94 | |
| Platelets, 10^3^/μL | 429 | | 364 | | 491 | | | 310 | | 56.90 | | | 0.14 | | 0.39 | |
| MPV, fL^5^ | 9.34 | | 8.79 | | 9.10 | | | 8.26 | | 0.44 | | | 0.38 | | 0.69 | |
| Total protein,  g/dL | 4.32^b^ | | 4.54^b^ | | 4.53^b^ | | | 5.19^a^ | | 0.19 | | | <0.05 | | 0.38 | |

^a,b^Within a row, means without a common superscript differ (*P* < 0.05).

^1^RBC = red blood cell, HGB = hemoglobin, HCT = packed cell volume, MCV = mean corpuscular volume, MCH = mean corpuscular hemoglobin, MCHC = mean corpuscular hemoglobin concentration, RDW = red cell distribution width, MPV = mean platelet volume, and PI = post-inoculation. Each least squares mean represents 12 observations, except d 11 PI that has 6 observations.

^2^LOW = Low dose blood group A6 type 1-based polymer (Coligo).

^3^HIGH = High dose blood group A6 type 1-based polymer (Coligo).

^4^CAR = carbadox.

^5^Linear effects of adding Coligo to the control diet.

^6^fL= femtolitre (10^-15^ L).
