# Supplementary material for: Supplementation of oligosaccharide-based polymer enhanced growth and disease resistance of weaned pigs by modulating intestinal integrity and systemic immunity
Source: J Anim Sci Biotechnol. 2022 Jan 12;13:10. doi: 10.1186/s40104-021-00655-2 (PMC8753815; doi:10.1186/s40104-021-00655-2)
Supplement: Supplementary file 3 — Additional file 3. Table S3 Intestinal morphology of ETEC-infected weaned pigs fed diets supplemented with oligosaccharide-based polymer (Coligo) or antibiotics. [file 40104_2021_655_MOESM3_ESM.docx]

**Table S3.** Intestinal morphology of ETEC-infected weaned pigs fed diets supplemented with oligosaccharide-based polymer (Coligo) or antibiotics

|  | Diet | | | | |  | | | | | | *P*-value | | | | |
| --- | --- | --- | --- | --- | --- | --- | --- | --- | --- | --- | --- | --- | --- | --- | --- | --- |
| Item^1^ | Control | | LOW^2^ | | HIGH^3^ | | | CAR^4^ | | SEM | | | Diet | | Linear^5^ | |
| d 5 PI^4^ |  | |  | |  | | |  | |  | | |  | |  | |
| Duodenum |  | |  | |  | | |  | |  | | |  | |  | |
| Villi height, µm | 396^b^ | | 488^a^ | | 494^a^ | | | 504^a^ | | 21.24 | | | <0.01 | | <0.01 | |
| Crypt depth, µm | 276 | | 296 | | 268 | | | 213 | | 27.25 | | | 0.17 | | 0.82 | |
| Villi height:Crypt depth | 1.48^c^ | | 1.72^bc^ | | 1.82^b^ | | | 2.42^a^ | | 0.15 | | | <0.01 | | 0.04 | |
| Villi width, µm | 130^b^ | | 154^a^ | | 150^a^ | | | 143^ab^ | | 5.62 | | | 0.04 | | 0.03 | |
| Villi area, µm^2^ | 55,360^b^ | | 78,230^a^ | | 71,410^a^ | | | 77,530^a^ | | 4,815 | | | 0.02 | | 0.03 | |
| Goblet cell number, per villi | 30.28 | | 33.18 | | 32.42 | | | 37.70 | | 2.19 | | | 0.19 | | 0.50 | |
| Sulfomucin area, % of villi area | 9.01 | | 8.59 | | 7.52 | | | 7.56 | | 0.75 | | | 0.42 | | 0.17 | |
| Sialomucin area, % of villi area | 5.42 | | 5.06 | | 5.29 | | | 4.34 | | 0.79 | | | 0.81 | | 0.91 | |
| Jejunum |  | |  | |  | | |  | |  | | |  | |  | |
| Villi height, µm | 379 | | 408 | | 447 | | | 449 | | 25.74 | | | 0.23 | | 0.09 | |
| Crypt depth, µm | 200 | | 197 | | 188 | | | 157 | | 17.78 | | | 0.40 | | 0.65 | |
| Villi height:Crypt depth | 1.92^c^ | | 2.14^bc^ | | 2.38^b^ | | | 2.91^a^ | | 0.17 | | | <0.01 | | 0.03 | |
| Villi width, µm | 106 | | 107 | | 110 | | | 113 | | 6.74 | | | 0.90 | | 0.67 | |
| Villi area, µm^2^ | 46,860 | | 54,300 | | 51,880 | | | 55,380 | | 5,786 | | | 0.75 | | 0.55 | |
| Goblet cell number, per villi | 14.86 | | 16.19 | | 18.85 | | | 18.20 | | 3.37 | | | 0.82 | | 0.40 | |
| Sulfomucin area, % of villi area | 6.72 | | 7.13 | | 5.69 | | | 4.67 | | 0.80 | | | 0.21 | | 0.38 | |
| Sialomucin area, % of villi area | 2.08 | | 2.40 | | 1.71 | | | 1.62 | | 0.40 | | | 0.38 | | 0.45 | |
| Ileum |  | |  | |  | | |  | |  | | |  | |  | |
| Villi height, µm | 326^b^ | | 345^b^ | | 377^a^ | | | 401^a^ | | 8.37 | | | <0.01 | | <0.01 | |
| Crypt depth, µm | 182 | | 159 | | 174 | | | 158 | | 10.93 | | | 0.17 | | 0.46 | |
| Villi height:Crypt depth | 1.84^c^ | | 2.19^b^ | | 2.23^b^ | | | 2.57^a^ | | 0.129 | | | <0.01 | | 0.02 | |
| Villi width, µm | 113 | | 106 | | 109 | | | 110 | | 5.96 | | | 0.86 | | 0.66 | |
| Villi area, µm^2^ | 37,790^b^ | | 39,230^b^ | | 46,520^a^ | | | 43,090^ab^ | | 2,846 | | | 0.08 | | 0.02 | |
| Goblet cell number, per villi | 19.15 | | 20.14 | | 22.06 | | | 18.97 | | 1.73 | | | 0.59 | | 0.25 | |
| Sulfomucin area, % of villi area | 7.91 | | 7.03 | | 6.17 | | | 5.41 | | 0.72 | | | 0.14 | | 0.10 | |
| Sialomucin area, % of villi area | 2.74 | | 2.32 | | 2.06 | | | 1.79 | | 0.42 | | | 0.48 | | 0.28 | |
| d 11 PI^4^ |  | |  | |  | | |  | |  | | |  | |  | |
| Duodenum |  | |  | |  | | |  | |  | | |  | |  | |
| Villi height, µm | 467^b^ | | 519^a^ | | 528^a^ | | | 523^a^ | | 16.14 | | | 0.06 | | 0.02 | |
| Crypt depth, µm | 232 | | 254 | | 247 | | | 221 | | 15.26 | | | 0.49 | | 0.50 | |
| Villi height:Crypt depth | 2.04 | | 2.10 | | 2.20 | | | 2.38 | | 0.12 | | | 0.27 | | 0.36 | |
| Villi width, µm | 150 | | 157 | | 160 | | | 148 | | 5.96 | | | 0.44 | | 0.24 | |
| Villi area, µm^2^ | 70,680^b^ | | 81,890^a^ | | 79,220^ab^ | | | 73,170^ab^ | | 3,203 | | | 0.07 | | 0.17 | |
| Goblet cell number, per villi | 31.85 | | 30.80 | | 31.44 | | | 35.24 | | 2.15 | | | 0.54 | | 0.90 | |
| Sulfomucin area, % of villi area | 7.32 | | 5.91 | | 8.64 | | | 7.97 | | 0.99 | | | 0.33 | | 0.36 | |
| Sialomucin area, % of villi area | 2.92^b^ | | 3.09^b^ | | 3.17^b^ | | | 5.99^a^ | | 0.76 | | | 0.05 | | 0.82 | |
| Jejunum |  |  | |  | | |  | |  | |  | | |  | |  |
| Villi height, µm | 396^c^ | | 444^b^ | | 500^a^ | | | 473^ab^ | | 11.70 | | | <0.01 | | <0.01 | |
| Crypt depth, µm | 198^ab^ | | 188^b^ | | 217^a^ | | | 174^b^ | | 10.10 | | | 0.04 | | 0.18 | |
| Villi height:Crypt depth | 2.03^b^ | | 2.40^ab^ | | 2.36^ab^ | | | 2.73^a^ | | 0.14 | | | 0.02 | | 0.08 | |
| Villi width, µm | 110 | | 112 | | 117 | | | 115 | | 4.36 | | | 0.69 | | 0.28 | |
| Villi area, µm^2^ | 50,450^b^ | | 52,540^ab^ | | 61,630^a^ | | | 54,650^ab^ | | 3,053 | | | 0.10 | | 0.02 | |
| Goblet cell number, per villi | 15.89 | | 16.71 | | 18.18 | | | 18.05 | | 1.47 | | | 0.67 | | 0.29 | |
| Sulfomucin area, % of villi area | 6.96 | | 5.53 | | 6.43 | | | 6.47 | | 0.57 | | | 0.40 | | 0.51 | |
| Sialomucin area, % of villi area | 1.72 | | 2.12 | | 2.10 | | | 2.33 | | 0.29 | | | 0.56 | | 0.37 | |
| Ileum |  | |  | |  | | |  | |  | | |  | |  | |
| Villi height, µm | 355^b^ | | 372^b^ | | 386^b^ | | | 427^a^ | | 12.48 | | | <0.01 | | 0.06 | |
| Crypt depth, µm | 162 | | 156 | | 174 | | | 169 | | 8.43 | | | 0.39 | | 0.29 | |
| Villi height:Crypt depth | 2.26 | | 2.39 | | 2.25 | | | 2.52 | | 0.12 | | | 0.41 | | 0.93 | |
| Villi width, µm | 110 | | 117 | | 120 | | | 105 | | 5.64 | | | 0.32 | | 0.24 | |
| Villi area, µm^2^ | 37,660^b^ | | 43,250^ab^ | | 47,580^a^ | | | 43,060^ab^ | | 2,566 | | | 0.09 | | 0.01 | |
| Goblet cell number, per villi | 22.86 | | 23.69 | | 25.41 | | | 20.48 | | 2.57 | | | 0.62 | | 0.49 | |
| Sulfomucin area, % of villi area | 6.61 | | 7.02 | | 6.49 | | | 5.28 | | 0.74 | | | 0.31 | | 0.89 | |
| Sialomucin area, % of villi area | 2.61 | | 2.37 | | 2.83 | | | 3.32 | | 0.38 | | | 0.40 | | 0.67 | |

^a,b,c^Within a row, means without a common superscript differ (*P* < 0.05).

^1^Each least squares mean represents 6 observations.

^2^LOW = Low dose blood group A6 type 1-based polymer (Coligo).

^3^HIGH = High dose blood group A6 type 1-based polymer (Coligo).

^4^CAR = carbadox.

^5^Linear effects of adding Coligo to the control diet.
